# On the Quality Inherent in the Inoculated Small-Pox of Propagating the Disease by Casual Infection

**Published:** 1811-08

**Authors:** 


### *To the Editors of the Medical and Physical Journal.*

### *On the Quality inherent in the Inoculated Small-Pox of propagating the Disease by casual Infection.*

" If it be true that the Practice of this Art has, for a series of years, augmented the Mortality of the natural Small-Pox : if, from the extension of that practice, a proportional increase of the mortality is to be apprehended, I cannot see on what principle, either of humanity or policy, the further use of it can be justified." WATKINSON.

GENTLEMEN,

I FIND by the Notices to Correspondents in your last number, p. 88, that a gentleman of extensive practical information thinks the observations published in your 148th Number, p. 490, with the Signature REPREHENSOR, unworthy of his notice because they are anonymous. I beg leave to say, that as the object of that letter was to shew, *by reference to authentic and acknowledged facts*, that the opinion of the non-infectious nature of the inoculated small-pox was untenable ; it mattered not whether the references were made under a real or a fictitious Signature ; for the facts referred to were to be judged by their own merits, and not from the name or the authority of the letter-writer.

It would indeed have sufficed, to refer to the memorable case recorded by Dr. Willan, or to the several cases published by Baron Dimsdale, which undeniably prove the infectious nature of the inoculated small-pox ; because few would be disposed to call in question a fact, affirmed by such competent judges as Baron Dimsdale, and Dr. Willan. But I was desirous of shewing further, that the power which the *mild* inoculated small-pox possesses, of communicating the casual infection, had been known and acknowledged, almost from the first introduction of inoculation into England.

To prove this, I referred to the testimony of Dr. Douglass, published ninety years ago ; seeing no reason to doubt the validity of his testimony, *as to this particular*.

I am told, however, in your notices to Correspondents, that the assertions of Dr. Douglass, on the contagious property of the inoculated small-pox, have *in themselves* " but little value." I think it therefore incumbent upon me, (without the least intention of defending his rudeness and illiberality) to substantiate his assertions by other authority, the credibility of which will hardly be impeached.

The first assertion of Dr. Douglass's which I have quoted, is, is, in effect, this; *that those who had maintained the non-infectious nature of the inoculated small-pox, were compelled by the evidence of facts to give up this tenet:* and I undertake to shew, that there is no good reason for disbelieving what he says, because there are facts upon record which warrant the assertion.

I appeal to the authority of Mr. Maitland as published by himself, in his "Account of inoculating the Small-pox," p. 26, Second Edition, 1723.

"October 2, 1721. After due preparation of the body I ingrafted Mary Batt, an infant of two years and a half old, daughter of Thomas Batt, a quaker, living at Temple, within three miles of Hertford. The red spots and flushings appeared on her face and neck the fourth day, and she kept playing about well till the seventh or eighth, when she became a little heavy and thirsty, with a fuller and quicker pulse; then the pustules came out fresh and full, and the incisions discharged a thick well digested matter. *She had not above twenty in all upon her;* they continued about three or four days, then dried away and fell off, and the child recovered perfectly.

"Thus far all was well; *but what happened afterwards was, I must own, not a little surprising to me, not having seen or observed any thing like it before.* The case was in short this; six of Mr. Batt's domestic servants, viz. four men and two maids, who all, in their turns, were wont to hug and caress this child whilst under the operation, and the pustules were out upon her, *never suspecting them to be catching, nor indeed did I,* were all seized *at once* with the right natural small-pox, of several and very different kinds; for some had the round distinct sort, some the small continued, and others the confluent: all of them had a great many, but especially the last, with the usual bad symptoms, and very narrowly escaped: but they all, God be thanked, did well, (except one maid, that would not be governed under the distemper, who died of it) and now enjoy a perfect state of health."

Mr. Maitland likewise, speaking of the inoculation of Mr. Heath's two sons, says, p. 28, "It is here also very remarkable, *further to evince the power of infection,* and the genuineness of the inoculated small-pox, that an infant of about four months old, then on Mrs. Heath's breast, while she nursed her two sons, and lay in bed with them, *was also seized with the distinct natural small-pox.*"

I am not ignorant, that when Maitland found the spreading of the natural small-pox through the town of Hertford charged upon this inoculation of Mr. Heath's sons, he endeavoured, in some measure, to explain away this his acknowledgment knowledgment of the infectious nature of the inoculated small-pox. For which purpose, he says in his "Account of inoculating the Small-pox vindicated from Dr. Wagstaffe's Misrepresentations," p. 25, second edition, 1722, "I own that it *seemed probable* that the six persons in Mr. Batt's family might have catched the small-pox of the girl that was inoculated: but it is well known that the small-pox were rife, not only at Hertford, but in several villages round it, many months before any person was inoculated there." He then enumerates several families who had the small-pox, and goes on to say; "besides all these, there were a great many more whose names I cannot at present call to mind, both in [the] town and [the] country about it, who had the small-pox, and several died of it, *the summer before* I began this practice.—To charge then the spreading the infection and the consequences of it, through that town, upon two single boys who were inoculated in a court in a manner separated from all the rest of the town," [by the bye, he says in the first account, p. 28, that one of these boys could not be kept within doors] "which was fuller of the small-pox before than after the inoculation, is not agreeable to that ingenuity which the Doctor (Wagstaffe) seems to demand of his adversaries."

Afterwards, p. 34, he says, "I think it is hard to exclude men from the means of securing themselves from a great pestilence, *upon a mere suggestion*;" from all which it seems probable that Mr. Maitland would have been very glad to have it believed, that the persons in question catched the small-pox from some other source, rather than from the inoculated disease. If, however, there were no other evidence that Mr. Batt's servants catched the infection from the inoculated child, (which Mr. Maitland now finds it convenient to admit to be *only probable*) the mere circumstance of their being "*all seized at once*," would go far to establish the fact.

But there is more complete evidence. An absurd suspicion was entertained, arising from the mild symptoms of the inoculated small-pox, that inoculation did not produce the genuine disease. To subvert this suspicion Mr. Maitland published the following certificate, as a complete proof that the real small-pox was given by inoculation, because from this identical *mild* small-pox, six persons had catched the genuine disease *in its several varieties* by casual infection.

"These are to certify, that Mr. Charles Maitland, Surgeon, did, about the beginning of October last, inoculate the small-pox upon my daughter Mary, aged two years and a half, *who had but few of them*, and perfectly recovered in about fifteen days. I do declare that six of my domestic servants were were seized with the small-pox, which, I believe, was owing to their carrying about and conversing with my said daughter, *they having had no correspondence during that time with any person or family who had them*, which inclines me to think my child had *the true small-pox*."

This certificate is not only signed by Mrs. Batt herself, but attested by the marks of "*two servants who received the small-pox from the child*," and who state that they "*know the contents to be true.*" A certificate to the same purport signed by Mrs. Heath was likewise published.

These extracts fully prove, that the *mildest* inoculated small-pox is infectious; and that Mr. Maitland and others were not aware of this fact, till it was *forced upon their conviction*, by the most indubitable evidence, and *the sacrifice of at least one poor woman to the variolous scourge*. This I think will lead us to acknowledge, that Dr. Douglass is worthy of credit, when he says, that the friends of inoculation at Boston "first gave out that it was a method not infecting," and that when "*many had died of the infection received from the inoculated, they gave up this point.*"

(*To be continued.*)

